# Supplementary material for: Effects of Contaminated Soil on the Survival and Growth Performance of European (Populus tremula L.) and Hybrid Aspen (Populus tremula L. × Populus tremuloides Michx.) Clones Based on Stand Density
Source: Plants (Basel). 2022 Jul 28;11(15):1970. doi: 10.3390/plants11151970 (PMC9370595; doi:10.3390/plants11151970)
Supplement: Supplementary file 1 [file plants-11-01970-s001.zip › plants-1665276-supplementary.pdf]

Supplementary Material (SM)

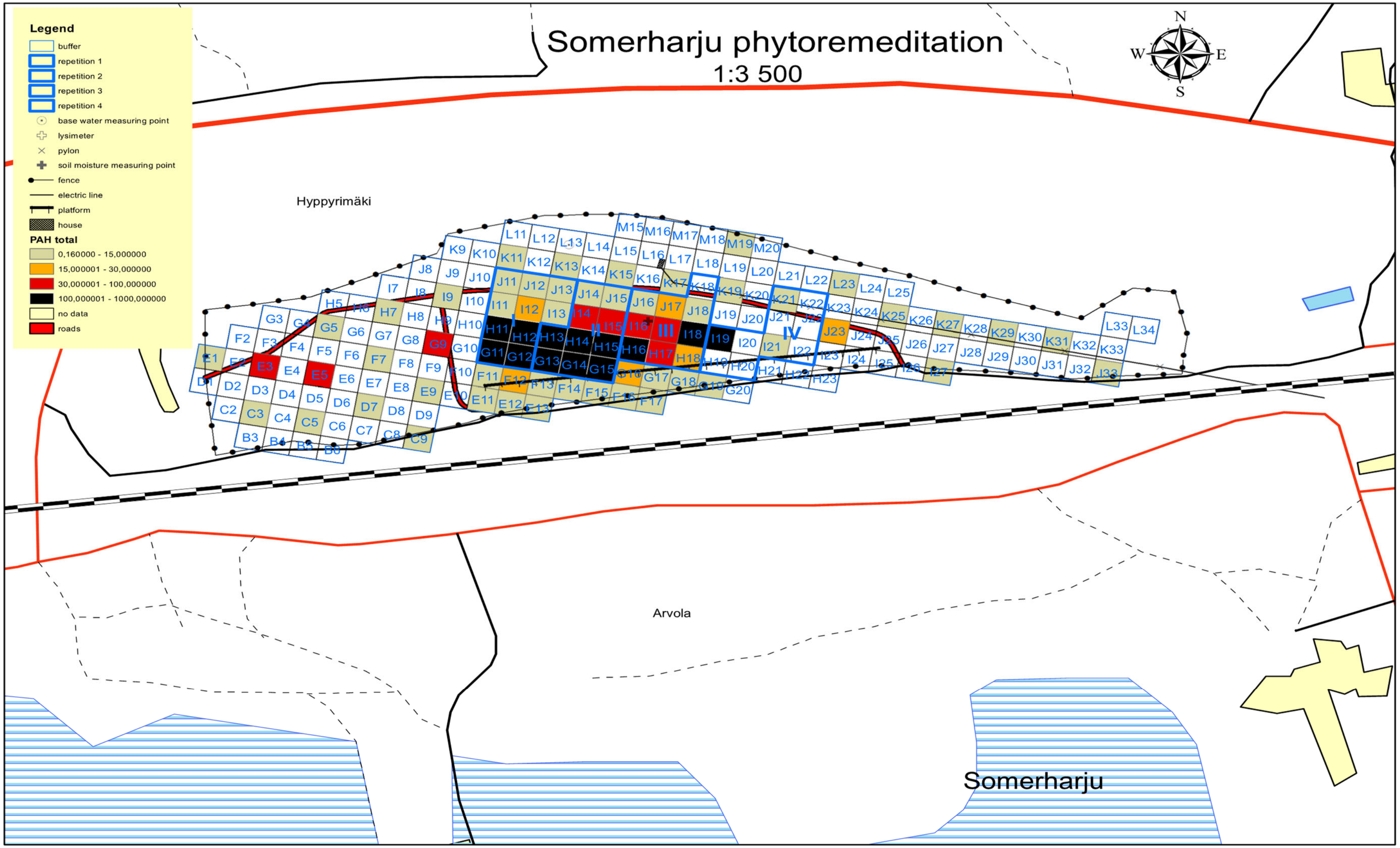

Figure S1. The experimental design of the Somerharju study area [37].

outside

N

greenhouse boarder

19	27 d1 old	R3 d6 old	291 d2 old
18	R3 d6 new	R3 d2 new	27 d6 new
17	R3 d2 old	R4 d2 new	R4 d2 old
16	R3 d2 clean	R4 d1 old	R3 d6 clean
15	R4 d1 clean	R4 d6 clean	291 d2 new
14	R3 d1 old	27 d1 new	291 d6 new
13	291 d2 clean	27 d6 clean	191 d2 new
12	14 d1 old	291 d1 new	27 d1 clean
11	R4 d6 old	14 d2 old	R4 d6 new
10	191 d6 new	14d2 clean	clean
9	new	191 d6 old	R4 d1 new
8	291 d6 old	291 d1 clean	191 d6 clean
7	191 d2 old	R3 d1 clean	191 d1 old
6	27 d6 old	R4 d2 clean	191 d2 clean
5	14 d1 clean	14 d6 new	14 d1 new
4	14 d2 new	291 d6 clean	14 d6 clean
3	191 d1 new	27 d2 old	R3 d1 new
2	27 d2 clean	old	191 d1 clean
1	291 d1 old	27 d2 new	14 d6 old
	1	2	3

Block 1

19	14 d2 old	14 d1 clean	R4 d6 old
18	R4 d1 new	R4 d2 new	R4 1 clean
17	R4 d2 old	R4 d1 old	R3 d1 clean
16	R3 d1 new	14 d1 new	R3d2 old
15	191 d2 new	291 d6 new	R3d1 old
14	R4 d6 clean	R3 d2 new	191 d6 old
13	191 d6 clean	291 d1 old	14 d6 clean
12	clean	R3 d6 clean	191 d2 clean
11	14 d6 new	191 d2 old	291 d2 new
10	191 d1 new	R3 d6 old	27 d2 new
9	27 d2 new	R4 d2 clean	27 d6 clean
8	14 d2 clean	27 d1 old	27d1 new
7	R4 d6 new	291 d1 clean	291 d2 clean
6	27 d6 new	27 d6 old	191 d6 new
5	14 d1 old	R3 d2 clean	old
4	14 d2 new	191 d1 clean	27 d1 clean
3	R3 d6 new	291 d6 old	291 d6 clean
2	new	291 d2 old	27 d2 old
1	291 d1 new	191 d1 new	14 d6 old
	1	2	3

Block 2

19	14 d1 clean	27 d2 new	27 d1 clean
18	14 d2 old	27 d6 old	291 d1 new
17	27 d1 old	14 d6 new	14 d2 clean
16	27 d2 clean	291 d2 new	R4 d1 new
15	R3 d2 clean	191 d6 old	27 d6 new
14	191 d1 old	R4 d1 old	R3 d1 new
13	27 d2 old	191 d2 clean	old
12	191 d1 new	R4 d2 new	291 d2 old
11	14 d6 clean	R4 d6 clean	14 d1 new
10	191 d2 new	R3 d1 clean	291 d6 old
9	14 d1 old	191 d2 old	R4 d1 clean
8	291 d6 new	27 d6 clean	new
7	14 d6 old	R4 d2 old	R3 d6 old
6	27 d1 new	191 d6 clean	R3 d1 old
5	191 d1 clean	14 d2 new	291 d6 clean
4	R3 d6 new	R3 d6 clean	R4 d6 new
3	291 d1 clean	R4 d2 clean	291 d2 clean
2	R4 d6 old	R3 d2 old	191 d6 new
1	291 d1 old	clean	R3 d2 new
	1	2	3

Block 3

central alley of the greenhouse

Figure S2. The layout of the experimental design for the greenhouse. Clean: pristine soil without contamination; new: pristine soil spiked with fresh diesel oil; old: creosote soil polluted with hydrocarbons; d: density (d1-low density, d2- medium density, d6-high density). Adapted from [7].

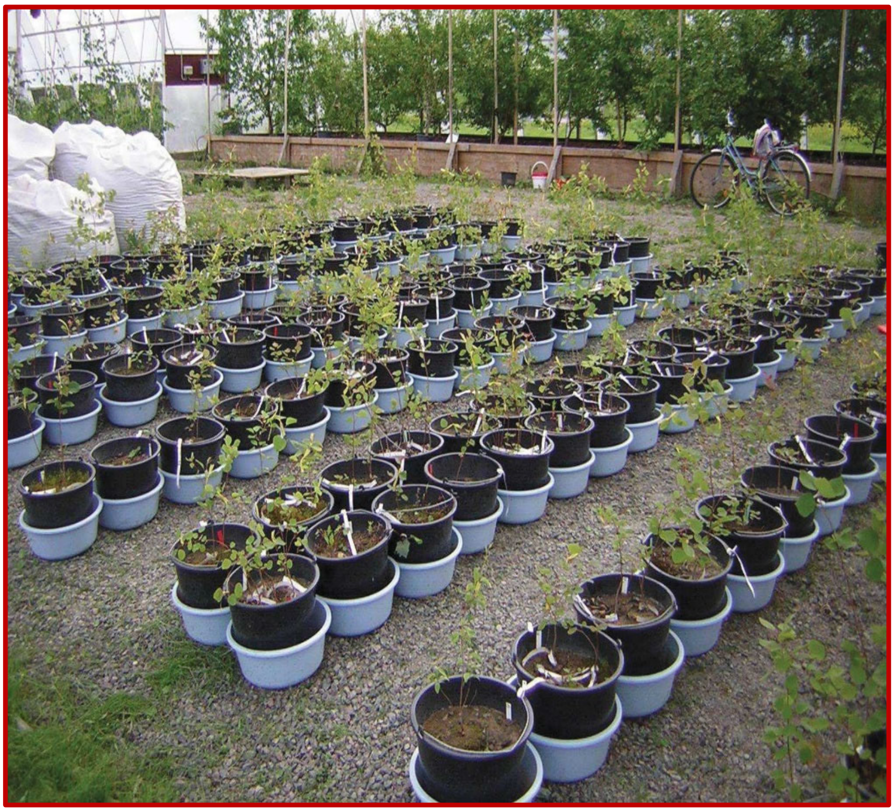

Figure S3. Plant's growth condition in the summer of 2015 at an open greenhouse, Haapastensyrjä tree-breeding center in Southern Finland (N: 60° 36´, E: 24° 25´). Adapted from [7].
